# A Semantic Medical Multimedia Retrieval Approach Using Ontology Information Hiding

**DOI:** 10.1155/2013/407917

**Published:** 2013-09-09

**Authors:** Kehua Guo, Shigeng Zhang

**Affiliations:** School of Information Science & Engineering, Central South University, Changsha 410083, China

## Abstract

Searching useful information from unstructured medical multimedia data has been a difficult problem in information retrieval. This paper reports an effective semantic medical multimedia retrieval approach which can reflect the users' query intent. Firstly, semantic annotations will be given to the multimedia documents in the medical multimedia database. Secondly, the ontology that represented semantic information will be hidden in the head of the multimedia documents. The main innovations of this approach are cross-type retrieval support and semantic information preservation. Experimental results indicate a good precision and efficiency of our approach for medical multimedia retrieval in comparison with some traditional approaches.

## 1. Introduction

In the past decades, the rapid growth of medical technologies has dramatically increased the amount of the multimedia data generated in various applications. The hospital will be swamped with medical multimedia documents such as images, videos, and audios. Medical multimedia retrieval has been a key technology in many medical applications. The research of solving some problems according to the features of medical multimedia retrieval attracts considerable attention. 

At present, medical multimedia retrieval is facing two problems. (1) Multimedia type diversity: in hospitals, the forms of medical multimedia documents are many, unstructured, and varied. Users may use various medical devices to generate images, videos, or audios. Even in image type, the data source may be computerized tomography (CT), magnetic resonance imaging (MRI), or positron emission tomography (PET). Type diversity makes it difficult to execute the cross-type search (e.g., searching a video according to an image). (2) Intent expression: in medical multimedia retrieval, the query intent generally can be represented by text. However, text can only express very limited query intent. Users do not want to enter too long text, but short text may lead to ambiguity. In many cases, the query intent may be described by content, but the content-based retrieval ignores the personal understanding because query intent information cannot be described by the physical visual data. Therefore, in the research of medical multimedia retrieval, how to search cross-type medical multimedia documents reflecting users' query intent has become more and more important.

Traditional medical multimedia retrieval approaches can be divided into text-based, content-based, and semantic-based retrieval [1]. In text-based approach, the search engine searches the text in the description file and returns the medical documents whose description contains the keywords typed by users. However, in hospitals, text description of the multimedia documents may be absent or even missing, and in this case, text-based query will be useless. In content-based approach, the search engine searches the files which are similar with the file uploaded by users using content-based method [2]. The main idea of content-based approach is feature extraction [3]. However, this approach cannot support cross-type search. In addition, the search engine will ignore the users' query intent and cannot get similar result satisfying users' search intent. In semantic-based approach, semantic features of medical multimedia documents are stored in the database for retrieval. However, if the medical multimedia document leaves the database, the retrieval process will not be able to perform unless the semantic information is rebuilt.

In this paper, our work develops a novel medical multimedia retrieval approach supporting cross-type retrieval and reflecting users' retrieval intent. The characteristics of our approach are as follows. (1) Cross-type retrieval support: we can arbitrarily upload image, video, or audio multimedia to obtain various suitable multimedia types. (2) Semantic information preservation: semantic information will not be lost even if the medical multimedia document leaves the medical multimedia database. Experimental results show that our approach can get a good performance.

The rest of this paper is as follows. The next section presents the related work of current medical multimedia retrieval approaches. In Section 3, we describe the system architecture, some key technologies, and performance evaluation model of our approach, respectively.  Section 4 illustrates the experimental results and the performance evaluation. Finally, we conclude the paper in Section 5.

## 2. Related Work

In fact, the retrieval approach for medical multimedia documents is similar to the common multimedia retrieval. The typical approach is focused on medical image retrieval [4], so (text-based image retrieval) TBIR and (content-based image retrieval) CBIR approaches have been widely used into the medical image retrieval. In the past decades, medical multimedia retrieval is mainly using text-based and content-based approaches. In text-based approach, the retrieval is only based on the text description. If the text does not exist, the retrieval cannot be executed. Therefore, the research of text-based approaches mainly concentrates on how to combine the text-based approach with other methods [5].

Although users feel more convenient to type text keyword, content-based retrieval approaches have been widely used [6, 7]. In system development, ASSERT system [8] used content-based approach for the retrieval of high resolution CT images of the lung. The authors of [9] proposed a system for the classification of medical images into anatomical areas and modalities. The authors of [10] gave a review on the generic content-based retrieval approach applied to medical image and described the techniques used in the system implementation, datasets design, and performance evaluations. However, it is very difficult to execute cross-type retrieval based on medical multimedia content. For example, for a video and audio multimedia document relevant to the same patient, we cannot identify the patient's identity or extract other similar features from the binary data of the two documents because their data formats are different. 

The approach proposed in this paper mainly uses semantic information to support medical multimedia retrieval. Multimedia retrieval reflecting users' query intent must fully consider the semantic information such as event, experience, and sensibility [11]. To solve this problem, some researchers used semantic annotation and user's feedback to improve the retrieval performance of content-based retrieval systems. For example, the authors of [12] collected the navigation data and visual features in CBIR system and adjusted them to adapt to the users' query intent. At present, the semantic information extraction approaches generally use the model of text semantic analysis. For example, the authors of [13] proposed a normalized cuts clustering algorithm to reduce the semantic gap. Many topic models such as (probabilistic latent semantic analysis) PLSA [14] and (latent dirichlet allocation) LDA [15] are widely used for the semantic extraction. In addition, (bag-of-words) BoW model [16] has been a typical model to express the visual words. 

In the field of unsupervised learning, some researchers combined relative feedback and machine learning. The authors of [17] used feedback and object model collaborative method to obtain the semantic information and get recognition results in a higher precision. In system development, the authors of [18] used (Hidden Markov Model) HMM to construct an automatic semantic image retrieval system. The authors of [19] investigated the ontology expression to virtual humans, covering the features, functions, and skills of a human. 

In summary, current research is mainly about the text and content-based approaches to medical image retrieval; these approaches cannot well support cross-type medical multimedia retrieval. Although the semantic analysis technologies have been widely used in some fields, they have not been used into the medical multimedia retrieval. In addition, in traditional semantic-based approaches, the isolation storage of the semantic information and multimedia data ignores the importance of semantic features. In this paper, we use the idea that saving semantic annotations together with the corresponding medical multimedia document can not only solve the retrieval dependence of semantic information on database but also support the cross-type medical multimedia retrieval. This idea has been used in [20] and indicated a good performance in ubiquitous multimedia retrieval.

## 3. The Proposed Framework

### 3.1. System Architecture

**Figure 1:**
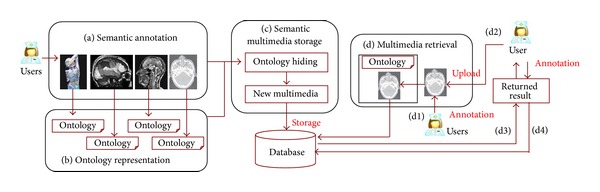
Architecture of our approach.

Our approach adopts a four-step architecture shown in Figure 1. The architecture mainly consists of semantic annotation (Figure 1(a)), ontology representation (Figure 1(b)), semantic multimedia storage (Figure 1(c)), and medical multimedia retrieval (Figure 1(d)) steps.

In the semantic annotation step, each medical multimedia document will be annotated by users in a hospital according to their personal understanding. The multimedia types may include images, videos, and audios with various formats and from various data sources. 

The text annotations provided by users will be represented by ontology technology in the second step. Our approach changes the annotations to ontology which is described based on tree structure. In the third step, the ontology representation will be saved together with the corresponding medical multimedia document using data hiding technology. After the new medical multimedia documents are generated, they will be saved into the medical multimedia database. 

The fourth step is retrieval process. In this step, user can upload an annotated medical multimedia document (Figure 1(d1)) with arbitrarily arbitrary format to execute the medical multimedia retrieval (Figure 1(d2)). In this case, the engine will return the result by matching the annotations of uploaded multimedia document with medical multimedia documents in database (Figure 1(d3)). 

In the returned result, the user will be asked to give additional annotations (Figure 1(d4)) to the medical multimedia he selected to make the annotations more abundant and accurate.

### 3.2. Semantic Annotation

In our approach, all the annotations will be described by text. We define *m* as a medical multimedia document and *C* as the set of medical multimedia documents satisfying *C* = {*m*_1_, *m*_2_,…, *m*_*N*_} (where *N* is the number of medical multimedia documents). For all *m*_*i*_ ∈ *C*, *m*_*i*_ will be saved in the hard disk of the server. The physical location information of *m*_*i*_ is saved in rational database linked to the corresponding real file. 

Semantic annotations will provide meaningful text reflecting users' personal understanding to *m*_*i*_. We define set *A*_*mi*_ as the annotation set of *m*_*i*_ satisfying *A*_*mi*_ = {*a*_1_, *a*_2_,…, *a*_*n*_} (where *n* is the number of annotations for *m*_*i*_). 

For arbitrary *m*_*i*_ ∈ *C*, users will give many annotations. However, not all the annotations can accurately represent the semantic information of *m*_*i*_. Therefore, for every *a*_*i*_ ∈ *A*_*mi*_, we assign a weight. For all  *m*_*i*_ ∈ *C*, the annotation matrix of *A*_*mi*_ is defined as

(1) $$\begin{array}{c} A_{mi}={\left[\begin{array}{c} a_{1},\ldots ,a_{n}\\ w_{1},\ldots ,w_{n} \end{array}\right]}^{T}, \end{array}$$

where *a*_*i*_ is the *i*th annotation and *w*_*i*_ is the corresponding weight. 

Therefore, all the annotation matrices for the medical database can be defined as *A* = {*A*_*m*1_, *A*_*m*2_,…, *A*_*mN*_}. For arbitrary *m*_*i*_ ∈ *C*, we assign the initial value of *w*_*i*_ is 1/*n*.

During the retrieval process, *w*_*i*_ for every annotation could not be constant. Obviously, more frequently used annotations during the retrieval process can better express semantic information, and they should be assigned a greater weight. We design an adjustment schema as follows:

(2) $$\begin{array}{c} w_{i}=w_{i}+k_{\text{i}}\times \frac{1}{n}, \end{array}$$

where *k*_i_ satisfies

(3) $$\begin{array}{c} k_{\text{i}}=\{\begin{array}{cc} 1 & m_{i}\;\;\text{is}\;\;\text{retrieved}\;\;\text{based}\;\mathrm{on}\;\;a_{i},\\ 0 & \text{others}. \end{array} \end{array}$$

The initial weight assignment and the adjustment process need to check all the medical multimedia documents in the database, and this work will cost many computational resources. To solve this problem, we can execute this process only once when the search engine is built. In addition, the adjustment process can be performed as background thread.

### 3.3. Ontology Representation and Semantic Multimedia Storage

We use ontology technology to describe the medical semantic information. In the ontology representation, each node describes one certain semantic concept and the ontology representation satisfies a recursive and hierarchical structure. Our approach adopts composite pattern [17] as the data structure to represent the relation of annotations.

In our approach, ontology semantic information will be merged with medical multimedia by two ways. (1) Online: in this approach, semantic annotations are submitted from software interfaces and saved together with the data of medical multimedia. (2) Offline: in this pattern, ontology semantic information is saved in a binary file whose extension name is “.s,” and the users can choose medical multimedia document to merge with the “.s” file. 

We utilize an optimal data hiding-based strategy for medical multimedia document storage. Our approach supports user feedback, and it may cause the modification of the semantic content, so we will design an effective approach to search and modify the semantic data in the medical multimedia. In our approach, we do not use some popular and security approaches such as neural network and wavelet technology and directly save the semantic information in the head of the medical multimedia. 

During every retrieval process, we cannot directly read and write medical multimedia documents in hard disk because this will cost lots of communication and computation time. To solve this problem, we adopt a cache-based approach. When the search engine is initialized, the semantic information in medical multimedia documents will be extracted to the rational database (e.g., Oracle, Microsoft SQL Server, etc.) for quick retrieval and maintained synchronization with the medical multimedia document data. This work will be executed in background thread. 

**Figure 2:**
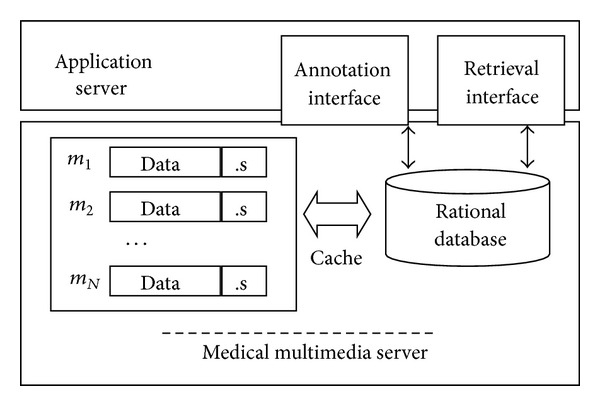
Multimedia server structure.

Client users visit the rational database through the annotation and retrieval interface, and then the medical server will find the real file of the medical multimedia.  Figure 2 shows the structure of the server which saved medical multimedia documents in the database. 

In Figure 2, “Annotation Interface” represents the software interface which provides annotations to the multimedia documents, and “Retrieval Interface” is the interface for uploading the multimedia document and submitting the retrieval requirement.

### 3.4. User Feedback

During the use of our approach, for arbitrary *m*_*i*_ ∈ *C*, the annotation matrix *A*_*mi*_ stems from the understanding of different users. The cardinality of *A*_*mi*_ will be more and more. In *A*_*mi*_, wrong or less frequently used annotations inevitably exist, which will waste much retrieving resource and storage space. In order to solve this problem, we define an optimization approach to eliminate the annotations which may be useless.

This process is called annotation refinement. The purpose is to retain most of the high frequency annotations and eliminate the annotations with less use. For arbitrary *m*_*i*_ ∈ *C*, the annotation refinement is described as follows.

(1) Check *A*_*mi*_ and remove the *i*th row when *a*_*i*_ satisfies
  (4) $$\begin{array}{c} w_{i}<\frac{1}{n}\sum_{i=1}^{n}w_{i}. \end{array}$$
(2) Rebuild *m*_*i*_.

Because this operation needs too much computation resource, we will execute the annotation refinement every long time interval and during the time of less retrieval requirements or system maintenance.

After retrieval, the engine will return some medical multimedia documents. Our approach supports user feedback, so for a particular returned multimedia document, the user can add additional annotations to enrich the semantic information. For these annotations, the initial weight will be 1/*n* too. 

In summary, during the retrieval progress, the annotations will be more and more abundant. But rarely used annotations will also be removed. There will be some new annotations added into the annotation matrix *A*_*mi*_ because of the user feedback. Therefore, our approach is a dynamic framework, which is used for the longer time and the more accurate results we can obtain.

### 3.5. Performance Evaluation

In this section, performance evaluation model will be designed to measure the performance of our approach. These models are based on the following five criteria: recall ratio, precision ratio, background process time cost, retrieval time cost, and additional storage cost.

(1)  *Recall Ratio and Precision Ratio.* The recall and precision ratios are the most common measurements for evaluating the retrieval performance. Now we use them to evaluate the performance of our approach. We can get different recall and precision ratios in different retrieval processes. For each retrieval process, we define the retrieved result set as *R*_*t*_ = {*m*_1_, *m*_2_,…, *m*_*t*_} (where *t* is the number of retrieved medical multimedia documents) and define all the relevant medical multimedia documents set as *R*_*l*_ = {*m*_1_, *m*_2_,…, *m*_*l*_} (where *l* is the number of relevant medical multimedia documents). 

The recall ratio is computed by the proportion of retrieved relevant medical multimedia documents in total relevant multimedia documents, and the precision ratio is computed by the proportion of retrieved relevant medical multimedia documents in total retrieval multimedia documents. Therefore, the recall ratio *R* and precision ratio *P* can be defined as follows:

(5) $$\begin{array}{c} R=\frac{M}{\left|R_{l}\right|},\\ P=\frac{M}{\left|R_{t}\right|}, \end{array}$$

where |#| represents the cardinality of a set and *M* is the number of relevant medical multimedia documents in the returned result and satisfies

(6) $$\begin{array}{c} M=\left|R_{t}\cap R_{l}\right|. \end{array}$$

It is an important issue to determine whether a returned multimedia document is relevant. In this paper, because the database is not very large, we will perform the judgment based on the users' understanding.

(2)  *Background Process Time Cost.* In our approach, several background processes will cost time. We define the background process time as follows:

(7) $$\begin{array}{c} T_{b}=T_{\text{mer}}+T_{\text{ref}}+T_{\text{cac}}, \end{array}$$

where *T*_mer_ is the merging time (merge the files with the semantic information) and *T*_mer_ = ∑_*i*=1_^*N*^ *T*_mer_^*i*^. *T*_ref_ represents the annotation refinement time (eliminate the redundant or error annotations) and *T*_cac_ represents the time cost of cache of the semantic information into the rational database. 

(3)  *Retrieval Time Cost.* We define *T*_*r*_ as the time cost for a special retrieval as follows:

(8) $$\begin{array}{c} T_{r}=T_{\text{ext}}+T_{\text{ret}}, \end{array}$$

where *T*_ext_ is the extraction time (extract the semantic information from the document), *T*_ret_ represents the retrieval and matching time.

(4)  *Additional Storage Cost.* Because the file size after merging will increase, the additional storage cost will be taken into consideration. The increase rate for storage *P*_*s*_ is defined as follows:

(9) $$\begin{array}{c} P_{s}=\frac{\sum_{i=1}^{N}\left(S_{\text{new}}^{i}-S_{\text{org}}^{i}\right)}{\sum_{i=1}^{N}S_{\text{org}}^{i}}, \end{array}$$

where *S*_new_ is the size of new medical multimedia documents and *S*_org_  is the size of original medical multimedia documents.

## 4. Experimental Results

### 4.1. Datasets and Experimental Tools

How to construct the dataset is an important problem in the experiment. Some general databases have been proposed. However, these databases can only perform the experiments aiming to one particular medical type (e.g., image files). Cross-type medical multimedia retrieval requires a wide variety of files such as images, videos, and audios, so these databases are not suitable to perform the experiments. 

We have constructed a medical database containing various medical types including images, videos, and audios. This medical database contains 10,000 medical multimedia documents, including 8,000 images, 1,000 videos, and 1,000 audios. All the annotations of the medical multimedia documents were provided through users manually annotating.

In this paper, we developed some software modules to verify the effectiveness of our approach. In the server, background process will be executed every 24 hours.  Table 1 shows the software tools and the running environment profiles in the experiments.

### 4.2. Recall and Precision Ratios

In the experiment, we choose a medical multimedia document (called sample document) and upload it to the search engine. The server will return the result by matching the annotations of uploaded multimedia document with medical multimedia documents in database. Before the search, some users will be asked to give the annotations to the sample multimedia document in the annotation interface. After uploading the file in retrieval interface, the system will search all the semantic medical multimedia documents whose semantic information is similar with the sample document. 

**Figure 3:**
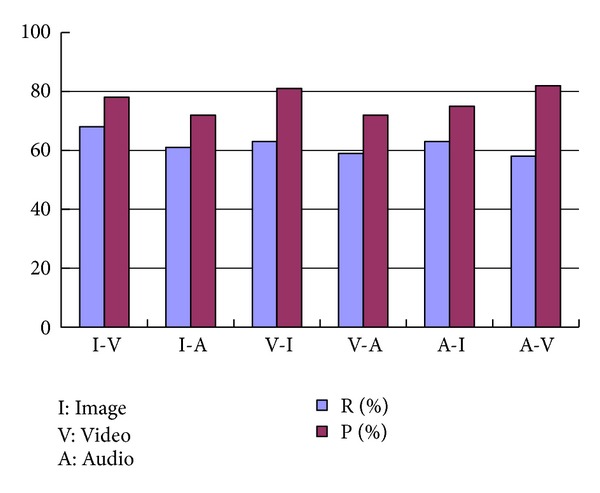
Average recall and precision ratios of retrieval.

To measure the performance, we use the images, videos, and audios as the sample files to execute the retrieval. In order to demonstrate the performance of the cross-type retrieval, we specially record the recall and precision ratios of using one type to search the other two types (e.g., using image to search videos and audios). To every multimedia type, we perform 10 different retrieval processes using 10 different sample documents and calculate the average recall and precision ratios to other multimedia types. The average recall and precision ratios are illustrated in Figure 3.

In Figure 3, T1-T2 represents searching T2 documents by uploading T1 type. Figure 3 indicates that in the retrieval process between different medical types, the recall and precision ratios are good. This is because our approach completely abandons the physical feature extraction and executes the retrieval processes based on semantic annotations which are described as text. 

### 4.3. Time Cost

In order to carry out the retrieval process, we have to perform several background processes whose time cost includes *T*_mer_, *T*_ref_, and *T*_cac_ defined in Section 3.  Table 2 shows the time cost of the first background processes. 

We can see from Table 2 that *T*_mer_, *T*_ref_, and *T*_cac_ will cost some seconds (*T*_*b*_ costs about 65 seconds for image type, 21 seconds for video type, and 18 seconds for audio type, resp.). However, the background processes are not always executed. In the server, background process will be executed every 24 hours in background thread, so this time cost can be acceptable.

Now we will measure the retrieval time *T*_*r*_ defined in Section 3. We specially record the time cost of 12 retrieval processes. We perform 4 different retrieval using 3 different multimedia types (image, video, and audio) which are numbered from 01 to 04. In every retrieval, *T*_ext_ and *T*_ret_ are recorded, respectively. The detailed time cost of retrieval of the 12 experiments is listed in Table 3.

Table 3 shows that the semantic information extraction only costs very short time, and this is because we only need to read the sample multimedia document and directly extract the semantic segment from it. After the extraction, the retrieval process will be similar with the text-based retrieval, and the table indicates that this process can be executed in acceptable time.

### 4.4. Storage Cost

The additional storage cost will be taken into consideration because the file size after merging will increase.  Table 4 shows the storage space cost before and after the information merging.

We can see from Table 4 that the file size after merging has almost not increased (*P*_*s*_ is about 0.39% for image type, 0.11% for video type, and 0.19% for audio type, resp.), and this is because the semantic information is represented as text and the size of semantic files is small.

## 5. Conclusions

In this paper, a new approach for medical multimedia document retrieval supporting cross-type medical multimedia retrieval and reflecting users' retrieval intent has been proposed. We designed the architecture of our approach and described semantic annotation, ontology representation, semantic multimedia storage, user feedback, and performance evaluation model. Experimental results show that our approach can achieve a good performance for the cross-type medical multimedia retrieval reflecting the users' intent.

The future work will concentrate on searching several improvements of our approach, including performing the experimentation in real medical environment and increasing the retrieval speed.

## Figures and Tables

**Table 1:** Software tools and running environment profiles.

Software tool	Development environment	Running environment
Annotation interface	Microsoft Foundation Classes (PC)	2.0 GHZ CPU, 1 GHZ RAM (PC)
Retrieval interface	HTML5 (Browser)	
Rational database	Oracle 11 g	Xeon E7-4820 2 GHZ, 16 GB RAM
Application server	Tomcat 6.0	

**Table 2:** Time cost of background processes (s).

Medical type	Quantity	*T*_mer_	*T*_ref_	*T*_cac_	*T*_*b*_
Image	8,000	27	18	20	65
Video	1,000	8	6	7	21
Audio	1,000	7	5	6	18

**Table 3:** Time cost of retrieval (ms).

Sample type	01		02		03		04	
	*T*_ext_	*T*_ret_	*T*_ext_	*T*_ret_	*T*_ext_	*T*_ret_	*T*_ext_	*T*_ret_
Image	43	1235	59	1129	68	1328	59	1025
Video	49	1628	61	1453	72	1952	67	1148
Audio	61	1365	68	1323	63	1775	62	1351

**Table 4:** Storage cost.

Document type	Quantity	∑_*i*=1_^*N*^ *S*_org_^*i*^ (MB)	∑_*i*=1_^*N*^ *S*_new_^*i*^ (MB)	*P*_*s*_ (%)
Image	8,000	17253	17321	0.39
Video	1,000	7895	7904	0.11
Audio	1,000	3624	3631	0.19
